# BedSect: An Integrated Web Server Application to Perform Intersection, Visualization, and Functional Annotation of Genomic Regions From Multiple Datasets

**DOI:** 10.3389/fgene.2020.00003

**Published:** 2020-02-05

**Authors:** Gyan Prakash Mishra, Arup Ghosh, Atimukta Jha, Sunil Kumar Raghav

**Affiliations:** ^1^Immunogenomics and Systems Biology Laboratory, Institute of Life Sciences, Bhubaneswar, India; ^2^School of Biotechnology, KIIT University, Bhubaneswar, India; ^3^Manipal Academy of Higher Education, Manipal, India

**Keywords:** web server tool, genomic region overlap, genomic colocalization, next generation sequencing (NGS), functional annotation

## Abstract

A large number of genomic regions, such as transcription factor binding sites (TFBSs) captured from next generation sequencing (NGS) data analyses or those available from the public resource database ENCODE, are generally overlapped to answer a variety of biological questions. Though several command-line tools are available to perform such an analysis, there is a notable lack of an integrated webserver application with which to identify genomic region intersections, generate publication-ready plots depicting subsets of the overlapped regions, and perform functional annotation. Thus, there is an ardent need for a comprehensive and user-friendly webserver application that allows the users to either upload multiple datasets or select from the integrated Gene Transcription Regulation Database (GTRD). We thus introduce BedSect (http://imgsb.org/bedsect/.), which not only fulfils the above criteria but also performs intersection analysis along with visualization of the intersection regions as an UpSet and correlation plot using the integrated Shiny application. Moreover, analyses, including functional annotation, gene ontology, and biological pathways enrichment for the identified unique and intersected genomic regions, can also be performed using the integrated GREAT tool. To view the genomic regions in the genome browser, the inbuilt hyperlink for UCSC can redirect the user to visualize the results as custom tracks.

## Introduction

Genomic regions of various interests, such as transcription factor binding sites (TFBSs), accessible chromatin regions, regions with histone modification, methylated regions, frequently interacting regions, and the like are accessible from several databases, such as the Encyclopedia of DNA elements (ENCODE) project (https://www.encodeproject.org) (Consortium, 2012), the Cistrome data browser (Zheng et al., 2019), etc. A recently developed gene transcription regulation database (GTRD) (Yevshin et al., 2019) compiles TFBSs and open chromatin regions by analyzing raw ChIPseq datasets form ENCODE and SRA. Genomic regions from multiple datasets are overlapped for better understanding and to make important biological interpretations and conclusions, such as identifying Hotspot regulatory regions bound by multiple TFs to understand gene regulation, identifying *de novo* DNA motif predictions at these sites, comparing DNaseI-hypersensitive sites (DHS) across different cells types, identifying conserved non-coding elements, etc. These binding events/chromosomal locations are stored generally in BED (browser extensible data) (Kent et al., 2002) format files. Many command line-based tools, such as BEDtools (Quinlan and Hall, 2010), BEDOPS (Neph et al., 2012), and Intervene (Khan and Mathelier, 2017) are available to overlap the BED files; however, these tools require knowledge of the linux command-line to carry out such an analysis. Though several R packages, such as ChIPpeakAnnon (Zhu et al., 2010), GUI, and web server tools, such as PeakAnalyzer (Salmon-Divon et al., 2010), PAVIS (Huang et al., 2013), and LOLA (Sheffield and Bock, 2016), are available to perform functional annotation of the genomic regions; the web servers, such as GSuite (Simovski et al., 2017) and Colo-Stats (Simovski et al., 2018), are also developed to perform a genomic regions comparison using genomic tracks, yet these tools come with limited functions of either overlaying only genomic regions, only visualization or functional annotation. At the same time the user is required to have some programming knowledge for using these tools. To considerably overcome these limitations, we developed a comprehensive web server application called BedSect (http://imgsb.org/bedsect/) that provides a platform for a genomic regions intersection of the users provided as well as a TFBSs BED file available from the integrated GTRD database, visualization of intersection regions such as UpSet plot (Lex et al., 2014), and correlation plot using an integrated Shiny application (ShinyApp). Moreover, it also generates individual BED files for unique as well as overlapping regions between the datasets that can be downloaded from the result page generated by the tool. Furthermore, the ShinyApp allows the user to generate publication-ready high-quality images for reporting. To perform functional annotation of these regions, an integrated link that uses the Genomic Regions Enrichment of Annotations Tool (GREAT)API (McLean et al., 2010) that performs annotation in terms of distribution of genomic regions with respect to transcription start site (TSS), GO enrichment, pathway enrichment, and enrichment against several other associated databases is needed. Also, the users can quickly use the links to redirect intersected or unique genomic regions in the University of California, Santa Cruz, (UCSC) genome browser for quick visualization of the results (Karolchik et al., 2007).

One of the important aspects in functional genomics is to identify genomic regions bound by a diverse set of transcription factors that are associated with histone modifications (either at promoter or enhancer regions) that mark changes in the chromatin structure (Chronis et al., 2017; Miller and Grant, 2013). To identify the distribution of such genomic regions near the TSS, and functionally annotate genes regulated by cis-regulatory elements bound by multiple regulatory factors, integration of publicly available webserver tools, such as the UCSC genome browser and GREAT, would be an immense advantage (McLean et al., 2010). PCR/ChIP-qPCR can be further performed to validate the predicted binding of different transcription factors once such regions are determined by BedSect. Integrated UCSC API for the purpose of visualizing the genomic regions as custom tracks in genome browser would therefore be quite helpful. As there is, as of yet, no such easy-to-use server, as an efficient user-friendly webserver tool to overlap multiple BED files, BedSect would be of great advantage to a broad audience working in the field of functional genomics.

### Design and Implementation

**Figure 1:**
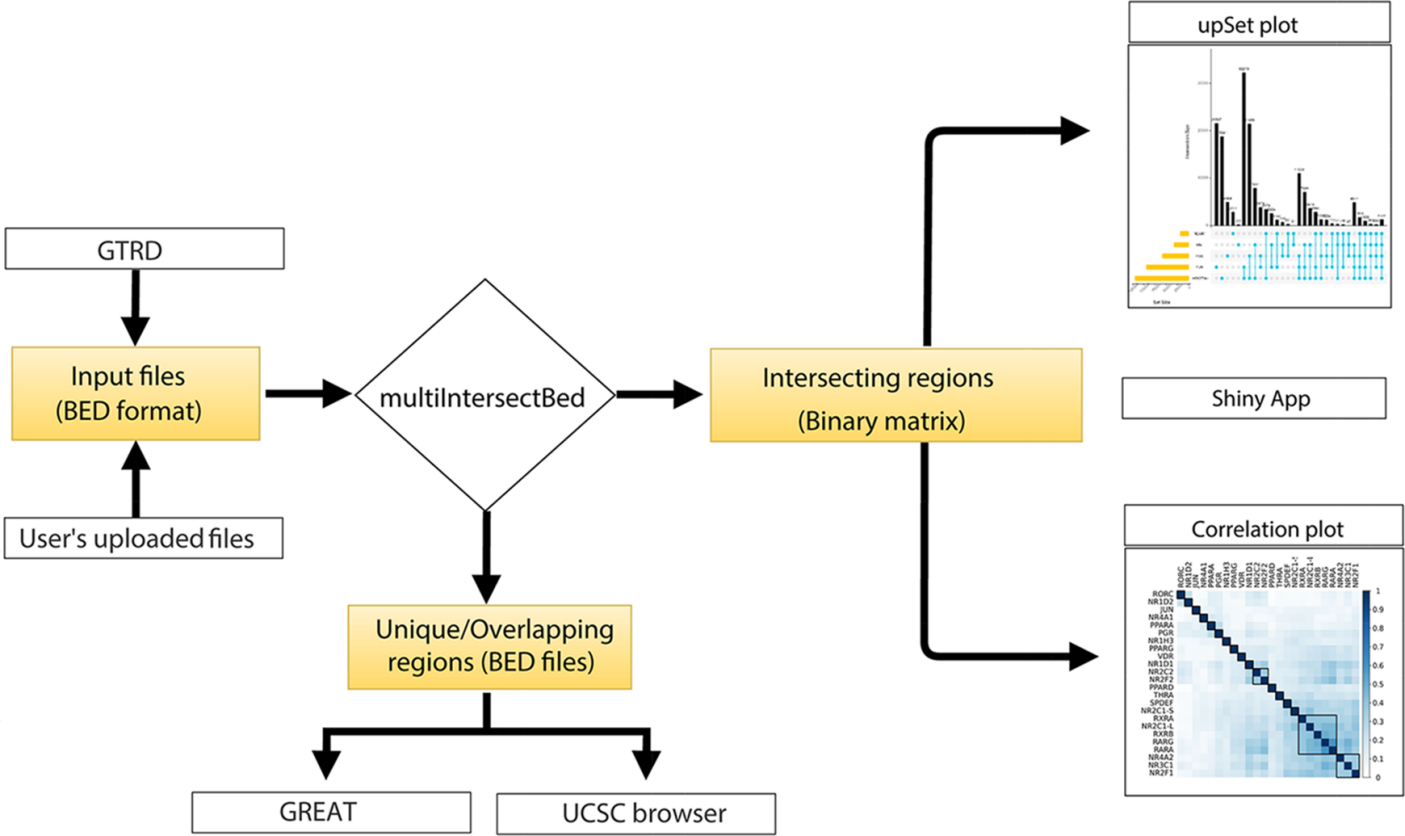
Flowchart representing the workflow of BedSect tool.

The core of the web server tool utilizes Perl, R, and the Shiny server, and the front end is based on PHP5.6, MySQL (Dist5.7.190) and Javascript 1.8. Genomic regions containing a file in a BED format can be provided by users or can be selected from the integrated GTRD database. All the processed BED files of TF ChIPseq peaks from the GTRD database for *Homo sapiens, Musmusculus, Daniorerio, Rattusnorvegicus, Drosophila melanogaster Scccharomycescerevisiae*, and *Schizosaccharomycespombe* were downloaded along with the metadata of experiments. Peaks called using MACS (Zhang et al., 2008) were further parsed to make a simplified three-column BED file. Once the input BED files are either uploaded or are selected from GTRD database by the users, the files are processed to find overlaps using the multiIntersectBed program of the BEDtools. Using a Perl parser script, the output file is parsed to extract overlapping regions between different datasets. The intersecting regions of interest can be narrowed down by the selection of the number of base pair overlaps provided by the users while submitting the files on the web server. The output generated from multiIntersectBed contains total genomic regions across all the datasets and its presence or absence across datasets are represented in terms of 0 and 1 (0 forabsenceand 1 for presence) in a binary matrix table. The matrix table is used to generate results that are represented by UpSet and correlation plots using integrated ShinyApp tool (**Figure 1**). To identify the similarities between different datasets, it calculates a pairwise Jaccard index between datasets from the obtained binary matrix and generates a correlation plot. Furthermore, to carry out functional annotation of the intersection regions, we integrated direct access to the GREAT server that annotates genomic regions to target genes and calculates statistical enrichment for the association of genomic regions (**Figure 1**). The default setting of a tool that annotates genomic regions to genes based on the distance to the TSS (proximal: 5kb upstream to 1kb downstream; distal; upto 1000kb) has been implemented, but if users intend to use other parameters, BED files of intersecting or unique regions can be downloaded and uploaded to the GREAT using desired settings. The tool also provides an option to hyperlink to submit unique or intersecting genomic regions as a custom track to be visualized in the UCSC genome browser (**Figure 1**).

### BedSect Web Server

**Figure 2:**
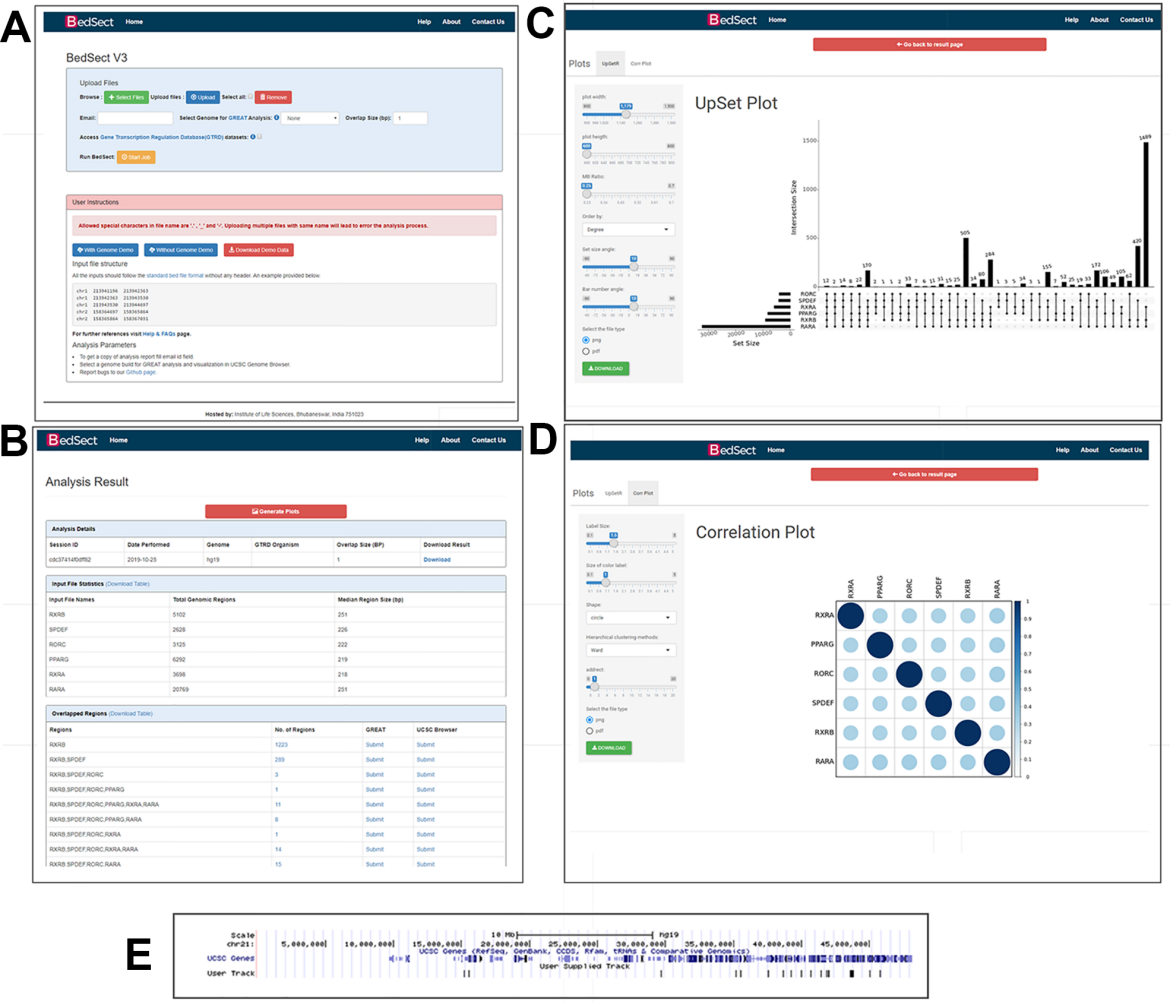
**(A)** Snapshot of the homepage of the web server. **(B)** Result page showing the analysis results after uploading the BED files. **(C)** UpSet plot representation of intersection regions. **(D)** Correlation plot representing similarity based on Jaccard indices (0 to 1). **(E)** Snapshot of UCSC genome browser showing uploaded user track.

BedSect's homepage features an input form with a set of fields, such as “browse files,” “genome,” “overlap size,” “email id,” “remove,” “upload,” and “start job,” that would help the users to use the tool effectively (**Figure 2A**). However, selecting the appropriate genome version for an uploaded file is necessary in to perform functional annotation and genome browser visualization. Depending on the availability of server resources as well as the number and size of files, the analysis may take several minutes; the email option will thus help to notify the status of the analysis, complete with links for access to BedSect analyzed results. After every successful submission, the user will be redirected to a results page (every 5 seconds) and, depending upon the status of the analysis, a specific message will be displayed. For two or more overlapping files, the user can select the BED format files that have the extension “.bed” using the “Select Files” tab and the “Upload” tab to upload files. To remove any uploaded files, the user can also select “Remove” tab to quickly remove the uploaded files. After a successful upload, the “Start Job” tab is used to start the analysis process. The user will receive an email for both job submission as well as for job completion. After completion of the analyses, a results page will display three tables (**Figure 2B**). The first table will show details of analysis submitted by the user along with “download” option to download the results files. The second table will display metadata providing total genomic regions and the median value of the genomic region size (bp), while the third table will provide individual rows for unique as well as intersecting regions along with links to submit the regions to the GREAT and the UCSC. Furthermore, customized and publication-ready plots that are easy to interpret can be immediately generated using the integrated ShinyApp. A “Tutorial” is available on the website to help the user become familiar with the usage of the web server whenever required.

### UpSet Plot

Other than a Venn diagram, one of the best alternatives to visualize subsets and sets is an UpSet plot. After a certain threshold, the interpretation of a Venn diagram becomes difficult; however, the BedSect tool provides an UpSet plots, which are easy to interpret. Moreover, the ShinyApp also provides a number of options to customize the generated plot (**Figure 2C**).

### Correlation Plot

To calculate the correlation between different datasets, we implemented the Jaccard function of an R package SuperExacttest (Wang et al., 2015), which calculates Jaccard statistics based on the total number of overlapping regions between datasets. The estimated values are then plotted as correlation heatmap using an R corrplot package (Wei, 2016). In addition, the ShinyApp provides the users with various options with which to customize the plot (**Figure 2D**).

### Functional Annotation and Visualization in the UCSC Browser

To analyze functional annotation and track visualization, the current version has been incorporated with the Mouse (mm10 and mm9 version), Human (GRCh38 and hg19 version), and Zebrafish (zv9 version) genomes. Upon selection of genome build at the home page, the result page provides a hyperlink option with which to submit the unique and intersecting regions to the GREAT server, and it also provides a further visualization in the UCSC browser (**Figure 2E**).

### Case Study

**Figure 3:**
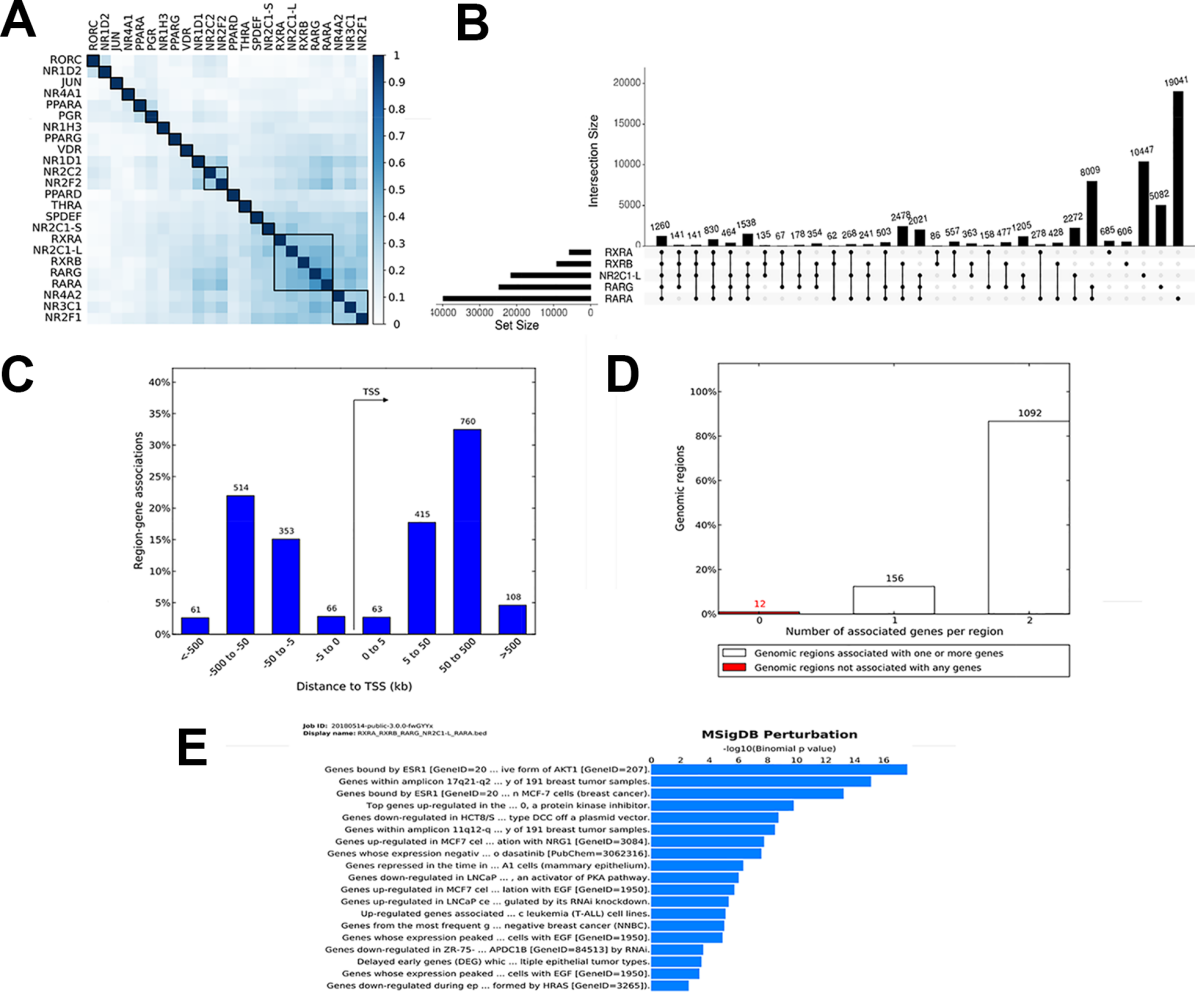
**(A)** Heatmap of correlation of 24 NR based on Jaccard index. **(B)** UpSet plot representing the number of subsets of intersection between five factors. **(C)** Genomic regions distribution near TSS. **(D)** Number of genes annotated to genomic regions. **(E)** Enriched pathway against MSigDB obtained from the GREAT analysis.

To demonstrate the utility of our tool, we studied the genome-wide binding of multiple transcription factors. Regions co-bound by multiple regulatory factors or histone modifications are considered HOTSPOTS that strongly regulate gene transcription. We downloaded online available datasets from a published study using the MCF7 breast cancer cell line, wherein 24 nuclear receptor (NR) bindings were profiled using NGS (GSE41995) (Chronis et al., 2017). The genome version of all the files was sourced from hg18 to hg19 using the liftOver tool from UCSC utilities. All the converted files are available at https://github.com/sraghav-lab/Bedsect/tree/master/test_data. It has been reported that the HOT regions (regions occupied by multiple transcription factors) play an important in cancer development (Chronis et al., 2017). To identify these regions, we implemented our comprehensive webserver application here, and we thus overlapped 24 NR peak files of the BED format. To identify factors that have a high genome-wide binding similarity, we generated correlation plots using the integrated ShinyApp by using the “Generate plots” tab. To identify a correlation, we used the Jaccard index to calculate the correlation and plotted the correlation plot using the R corrplot package. We demonstrated that RARα, RARγ, RXRα, RXRβ, and NR2C1-L bindings are highly similar based on genome-wide occupancy of all these factors (**Figure 3A**). A recent study published by *Kittler et al*. has shown, using network analysis, that RARα, RARβ, RXRα, and RXRβ regulate genes associated with breast cancer development (Kittler et al., 2013). We then uploaded BED files of only these five datasets to identify regions co-bound by only these five transcription factors. In addition, to identify the distribution of the overlapped regions near the TSS, we submitted the identified regions (n = 1262) co-bound by these five trans-factors to the GREAT (**Figure 3B**). Interestingly, we found that the majority of the bindings were present in far-distal regions (> 5kb to TSS) (**Figure 3C**). The gene annotation plot showed that 12 regions were not annotated to any gene, 156 regions were annotated to one gene, and each of the 1092 genomic regions were annotated to two genes (**Figure 3D**). Furthermore, to predict the functional role of the genes annotated to intersected genomic regions or HOT regions, we looked into the enriched pathway against MSigDB. The enriched pathways indicated an association of annotated genes with breast cancer, gastrointestinal tumors, as well as an epithelial—mesenchymal transition (**Figure 3E**). Thus, the test analysis of the presented datasets suggests that the BedSect tool is an efficient and powerful tool with which to identify regions intersecting between different datasets and aids the rapid prediction of the functional importance of the intersected genomic regions.

### Comparison With Other Publicly Available Tools

To show the advantages and usefulness of our tool, BedSect, we compared it with various publicly available tools based on various attributes. **Table 1** shows the comparison of BedSect with all other currently available tools.

**Table 1:** Comparison of BedSect with other publicly available tools.

Tools	Algorithm/Methods	Generate figures (type)	Intersection regions file as output	Functional annotation	Visualization in genome browser	Platform type or Utility
BEDtools (Quinlan and Hall, 2010)	Set theory on the genome	×	✓	×	×	Command line based for genomic regions overlap
Pybedtools (Dale et al., 2011)	Classical Venn	✓ (Venn diagram)	✓	×	×	Command line based for genomic regions overlap
ChippeakAnno(Zhu et al., 2010)	Classical Venn	✓ (Venn diagram)	✓	×	×	R package
PAVIS (Huang et al., 2013)	Functional annotation	✓ (Pie Chart)	×		×	Web server only for functional annotation
PeakAnalyzer(Salmon-Divon et al., 2010)	Functional annotation	(Bar plot for Genomics region Distribution to TSS)	×		×	Command line based for functional annotation and genomic regions overlap of two genomic region files.
regioneR(Gel et al., 2016)	Randomization-based	×	×	×	×	R package
						
BEDOPS(Neph et al., 2012)	Suite	×	✓	×	×	Command line Based for genomic region intersection
Intervene (Khan and Mathelier, 2017)	Classical Venn, Euler, Edwards, Chow-Ruskey, Square, Battle	✓ (Venn diagram, UpSet plot, Correlation heatmap)	✓	×	×	Command line based for genomic regions intersection and visualization. Web server only for visualization
BedSect	Integration of multiple tools (BEDtools, GTRD GREAT, UCSC, ShinyApp)	✓ (UpSet plot, Correlation heatmap)	✓	✓	✓	Completely Web server based for the intersection, visualization and functional annotation

## Data Availability Statement

We downloaded online available datasets from a published study using MCF7 breast cancer cell line, where 24 nuclear receptors (NR) bindings were profiled using NGS (GSE41995). The tool is available at http://imgsb.org/bedsect/ and source code is available at Github (https://github.com/sraghav-lab/Bedsect).

## Author Contributions

GPM, SR, and AG conceived and designed the project. AG and GPM designed the tool. SR supervised the project. GPM wrote the manuscript, and AG, AJ, and SR provided suggestions for improvement. All authors read and approved the manuscript.

## Funding

This work has been supported by grants from the DST-SNSF (DST/INT/SWISS/SNSF/P-47/2015), DBT Ramalingaswami fellowship, SERB (EMR/2016/000717), DBT (BT/PR15908/MED/12/725/2016), and the Institute of Life Sciences, Bhubaneswar, provided intramural support and infrastructure. GM is supported by the DBT- BINC JRF fellowship, and AG and AJ are supported by the institutional fellowship program.

## Conflict of Interest

The authors declare that the research was conducted in the absence of any commercial or financial relationships that could be construed as a potential conflict of interest.
